# Effect of 8-Day Fasting on Leukocytes Expression of Genes and Proteins Involved in Iron Metabolism in Healthy Men

**DOI:** 10.3390/ijms22063248

**Published:** 2021-03-23

**Authors:** Andżelika Borkowska, Maja Tomczyk, Małgorzata Żychowska, Wiesław Pilis, Michał Zych, Jędrzej Antosiewicz

**Affiliations:** 1 Department of Bioenergetics and Physiology of Exercise, Medical University of Gdansk, 80-210 Gdansk, Poland; andzelika.borkowska@gumed.edu.pl; 2 Department of Bioenergetics and Nutrition, Faculty of Physical Education, Gdansk University of Physical Education and Sport, 80-336 Gdansk, Poland; maja.tomczyk@awf.gda.pl; 3 Department of Sport, Faculty of Physical Education, Kazimierz Wielki University in Bydgoszcz, 85-064 Bydgoszcz, Poland; mz0511@ukw.edu.pl; 4 Department of Health Sciences, Jan Długosz University in Częstochowa, 42-200 Częstochowa, Poland; w.pilis@ujd.edu.pl (W.P.); m.zych@ujd.edu.pl (M.Z.)

**Keywords:** ferritin, PCBP, ferroportin, transferrin receptor, APP

## Abstract

The popularity of fasting and restricted food intake is increasing. While the body’s adaptability to dietary insufficiency is crucial for health, molecular mechanisms of adaptive changes are not well understood. Here, we compared the effects of fasting and exercise on the expression of leukocyte genes and proteins involved in the storage, export, and acquisition of iron, an essential element with physiological roles. Healthy men participated in the study (age, 30–70 years; body weight, 60–100 kg; body mass index, 20–29.9 kg/m^2^). The participants performed an exercise test with a gradually increasing intensity until the individual maximum exercise capacity was reached, before and after 8-d fast. Blood samples were collected before, immediately after, and 3 h after exercise. Gene expression was analyzed by reverse-transcription quantitative polymerase chain reaction and protein levels were analyzed by immunobloting. Eight days of total starvation diet affected the body composition and decreased exercise capacity. Further, fasting decreased the expression of genes associated with iron storage and export, and increased the expression of genes involved in iron acquisition. Conversely, only PCBP2 protein increased after fasting; however, an upward trend was apparent for all proteins. In conclusion, the body adapts to starvation by adjusting iron economy.

## 1. Introduction

Periods of starvation were natural for human ancestors. Indeed, calorie restriction is associated with improved health, weight reduction, increased insulin sensitivity, diabetes control, or cancer prevention [1]. The human organism is able to adjust to dietary insufficiency both at the physiological and biochemical levels, allowing survival during starvation periods. During starvation, iron metabolism is reduced to internal iron turnover. Maintaining appropriate iron levels is crucial for the human body because iron participates in multiple metabolic pathways [2]. Human organism possess complicated mechanism to respond to iron deficiency [3,4]. 

In conditions of iron deficiency synthesis of proteins responsible for iron incorporation into the cell is activated. These proteins include the transferrin receptor (TFRC) and divalent metal transporter (DMT1). Cellular iron is then used up and its excess stored in ferritin. Ferritins (FTH and FTL) play a crucial role in iron storage and play important antioxidative role by regulating the labile iron pool (LIP). In addition, chaperone proteins, human poly(rC)-binding proteins (PCBPs 1–4) bind iron; however, the physiological role of these proteins is not fully understood. In mammalian cells, only PCBP1 and PCBP2 are expressed at high levels [5]. According to some studies, PCBP2 directly interacts with DMT1 and facilitates the transfer of iron from this protein to the cytosol [6]. Furthermore, PCBP2 binds to ferroportin and promotes the efflux of cytosolic iron [7]. In 2008, PCBP1 was identified as an iron chaperone, that deliver iron to ferritin [8]. According to Leidgnes et al. (2013), each PCBP exhibits iron chaperone activity toward ferritin [9]. Iron chaperone activity of PCBP1 is essential for cell physiology. In the reticulocytes, depletion of PCBP1 leads to defects in the delivery of intracellular iron to ferritin, which in turn results in defects in iron incorporation into heme [10]. In recent years, it has also been shown that APP, a protein most often associated with Alzheimer’s disease, is involved in the export of iron out of the cell. APP facilitates iron export by stabilizing ferroportin at a hepcidin-binding site, which stimulates ferroportin degradation [11,12].

Despite notable interest in the changes in the body elicited by fasting, data on the changes in iron metabolism are not conclusive, and mostly pertain to alterations in serum or plasma iron levels. For example, Afolabi et al. (2007) showed that 5 d of total starvation exerts only a small effect on iron serum levels and plasma transferrin levels. Similarly, a 2-d fast does not significantly affect iron levels in human. By contrast, in women who starved for 2 d over an 8-d period in a 48-d study, a significant decrease of serum and hair iron levels was observed, as well as a decrease of ferritin and hemoglobin (Hb) levels, hematocrit (Hct), red blood cells, and total iron binding capacity [13]. The above changes appear to be dependent on the starvation duration. 

Fasting leads to a body energy deficit and should reduce the exercise capacity. Maintaining the appropriate level of iron is important because of its role in oxygen transport via Hb and myoglobin, and in energy production, which highlights its crucial role in physical activity [2]. Moreover, there are some data showing that changes in iron metabolism modulate inflammatory response [14,15,16,17]. Therefore, it became evident that iron regulates immunometabolism and that its impairment contributes to alterations in the immune system [18]. Conversely, it has been suggested that cells of the immune system are better adapted to changes in iron supplies than cells of other tissues [19]. Thus, leukocytes are the cells in which problems with iron economy should appear relatively late when organism’s metabolism is impaired. Moreover, no correlation between different iron markers and the differential white blood cell counts in African school children was reported [20]. 

Previous study on animal model showed that prolonged fasting could activate hypoxia inducible factor (HIF-1 alpha) [21]. This transcriptional activator is involved in the adaptive response to hypoxia [22] and is able to control expression of over 60 genes directly by hypoxia response elements (HRE) [23]. Among genes sensitive to HIF-1a are the genes associated with iron homeostasis and energy production, such as: transferrin (Tf), transferrin receptor (TfR), ferroportin, iron regulatory protein-1 (IRP1) or genes encoding glycolytic and lipolysis enzymes. Iron regulation during hypoxia is also dependent on HIF-2 activity, which can be directly sensitive for iron through 5′ iron responsive element (IRE), which may indicate its important role in iron metabolism [24].

Independent of fasting, acute physical activity affects iron and iron-regulatory protein status by increasing oxidative stress and inflammation [25,26,27]. In a study in a rat model, opposite effects of fasting and exercise on the morphological parameters of the blood, lipid metabolism, and activity of blood Na,K-ATPase were reported [28]. However, to the best of our knowledge, no data are available on the effects of starvation on the expression of intracellular proteins and genes involved in iron metabolism in white blood cells, and the impact of these effects on exercise capacity. We here investigated the changes in leukocyte iron metabolism after 8-d fasting, and the effects on exercise capacity of adult men. We anticipated that 8-d fasting would increase or decrease protein and mRNA levels responsible for iron acquisition and export respectively, as well as exercise capacity.

## 2. Results

Table 1 summarizes data on somatic and selected biochemical indicators before and after 8-d of fasting. Significant changes were observed in all somatic parameters after 8-d fasting. Decrease in body mass was associated with a decrease in fat mass, fat-free mass (FFM), and total body water (TBW). However, the effect size was moderate for all parameters except for fat mass (effect size small). At the same time, β-hydroxybutyrate level significantly increased (effects size large). Hb and HCT decreased, and changes in these indicators (not significant, effect size for Hb large) could have been related to an increased circulating blood volume (Table 1).

### 2.1. Gene Expression and Oxidative Stress

#### 2.1.1. Effect of 8 Days Starvation on Gene Expression

**Figure 1:**
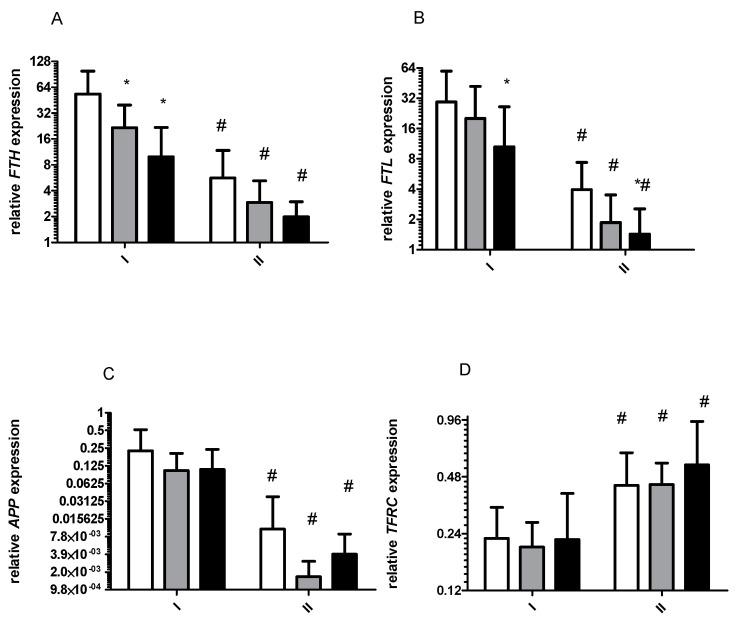
Response to physical effort on the gene expression level before (I) and after (II) 8 d of total starvation diet. Normalized expression of the following genes is shown: (**A**), *FTH*; (**B**), *FTL*; (**C**), *APP*; (**D**), *TFRC*. Before exercise, white bars; immediately after exercise, gray bars; 3 h after exercise, dark bars. Data are mean ± SE (n = 13). Analysis of each samples were done in triplicate. * Significant difference (*p* ˂ 0.05), baseline vs. post-effort. ^#^ Significant difference (*p* ˂ 0.05), I vs. II at the same time point. All mRNA levels are expressed as log2 expression relative to *TUBB*.

**Figure 2:**
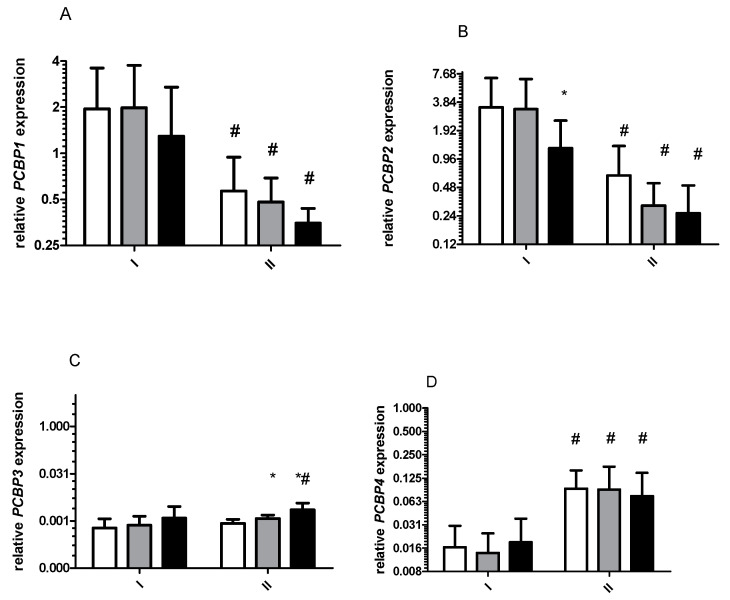
Response to physical effort on the gene expression level before (I) and after (II) 8 days of total starvation diet. Normalized expression of the following genes is shown: (**A**), *PCBP1*; (**B**), *PCBP2*; (**C**), *PCBP3*; (**D**), *PCBP4*. Before exercise, white bars; immediately after exercise, gray bars; 3 h after exercise, dark bars. Data are mean ± SE (n = 13). Analysis of each samples were done in triplicate * Significant difference (*p* ˂ 0.05), baseline vs. post-effort. ^#^ Significant difference (*p* ˂ 0.05), I vs. II at the same time point. All mRNA levels are expressed as log2 expression relative to TUBB.

**Figure 3:**
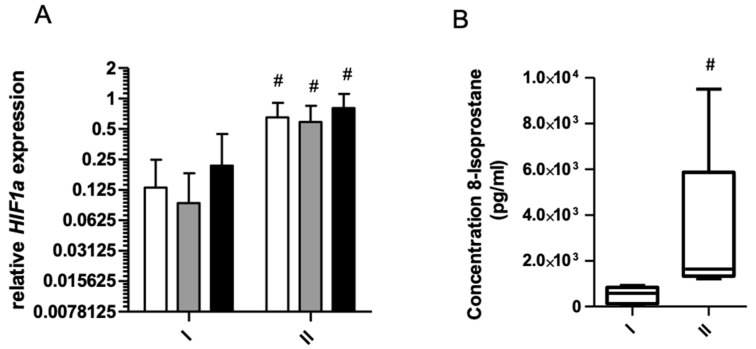
(**A**), Response to physical effort on the gene expression level before (I) and after (II) 8 days of total starvation diet. Normalized expression of *HIF1a* is shown. Before exercise, white bars; immediately after exercise, gray bars; 3 h after exercise, dark bars. Data are mean ± SE (n = 10). Analysis of each samples were done in triplicate ^#^ Significant difference (*p* ˂ 0.05), I vs. II at the same time point. All mRNA levels are expressed as log2 expression relative to TUBB. (**B**), Change in serum 8-isoprostane concentration level before (I) and after (II) 8 days of total starvation diet. Analysis of each samples were done in duplicate. ^#^ Significant difference (*p* ˂ 0.05), I vs. II at the same time.

*Ferritins* and *APP* mRNA levels decreased significantly after starvation period (Figure 1). These changes were observed at rest, as well as immediately and 3 h after exercise (Figure 2A,B). Similar changes were noted for *PCBP1* and *PCBP2* mRNA (Figure 2A,B). The opposite effect was observed for *TFRC* (Figure 1D, of *HIF1a* (Figure 3A) and *PCBP4* mRNA (Figure 2D) i.e., their levels significantly increased after starvation at all time points and only 3 h after exercise, respectively (Figure 2C). Two-way ANOVA revealed significant differences in expression of all analyzed genes between investigated time points. 

#### 2.1.2. Effect of 8 Days starvation on 8-Isoprostane

Significant increase in 8-isoprostane a marker of lipid peroxidation was observed after fasting (Figure 3B).

#### 2.1.3. Effects of Exercise on Gene Expression before and after 8 Days of Starvation

Before starvation, exercise caused a decrease in *FTH* and *FTL* mRNA levels (Figure 1A,B). In detail, a significant decrease was observed for *FTH* mRNA immediately after (*p* = 0.01) and 3 h after exercise (*p* = 0.006) while *FTL* mRNA levels significantly decreased 3 h after exercise (*p* = 0.048). Minor changes in *APP* and *TFRC* mRNA levels were noted at the same time.

After starvation, exercise caused a significant decrease in *FTL* mRNA level 3 h from the end of the effort (*p* = 0.05) (Figure 1B) and a significant increase in PCBP3 mRNA (*p* = 0.03) immediately after the effort. No effects of exercise were observed for other tested genes.

### 2.2. Immunoblotting

**Figure 4:**
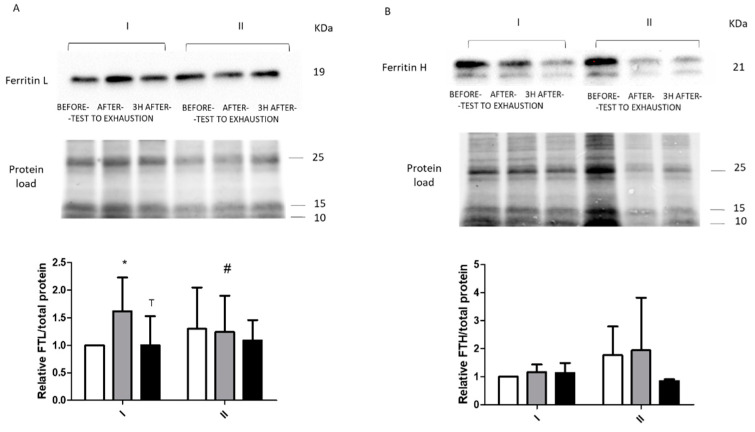
Response to physical effort of ferritin proteins. Representative immunoblot of ((**A**), FTL) and ferritin H ((**B**), FTH) levels before (I) and after (II) 8 d of total starvation diet. Differences in protein loading were normalized to total protein using stain-free gels. Data are mean ± SE of data for 10 subjects (FTL) or 9 subjects (FTH). * Statistical significance (*p* ˂ 0.05), baseline vs. post-effort. Analysis of each samples were done in single. ^#^ Statistical significance (*p* ˂ 0.05), I vs. II at the same time point. ^T^ Significant differences (*p* ˂ 0.05), immediately after vs. 3 h after the physical effort.

**Figure 5:**
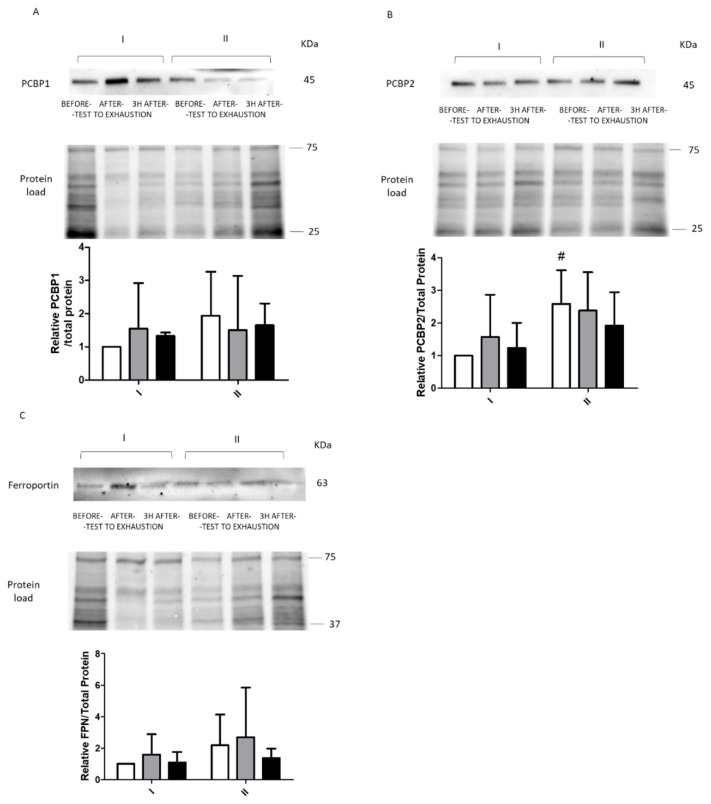
Response to physical effort of ferroportin and iron chaperons. Representative immunoblot of PCBP1 (**A**), PCBP2 (**B**), and ferroportin ((**C**), FPN) levels before (I) and after (II) 8 d of total starvation diet. Differences in protein loading were normalized to total protein using stain-free gels. Bar graphs present the mean ± SE of data for 9 subjects (PCBP1), 6 subjects (PCBP2), and 7 subjects (FPN). Analysis of each samples were done in single ^#^ Statistical significance (*p* ˂ 0.05), I vs. II at the same time point.

Changes in the intracellular levels of tested proteins were largely not statistically significant. However, exercise before fasting resulted in a significant increase in FTL levels immediately after the exercise (from 1 to 1.62, *p* = 0.02) and a significant decrease 3 h after the exercise (from 1.62 to baseline, 1.00, *p* = 0.003). No such changes were observed for exercise performed after 8 d of total starvation diet. Exercise before fasting did not affect FTH levels. Interestingly, despite the lack of statistical significance, an upward trend was noted for both ferritin levels in response to fasting (FTL, from 1 to 1.30; FTH, from 1 to 1.77) (Figure 4). We also noticed a similar tendency for other proteins (PCBP1, PCBP2, and FPN). However, statistical significance was only noted for PCBP2 at baseline (from 1 to 2.59, *p* = 0.03) (Figure 5). Furthermore, significant changes immediately after exercise before and after fasting were observed in FTL levels (a decrease from 1.62 to 1.24, *p* = 0.047).

## 3. Discussion

In the current study for the first time we demonstrate that 8-d fasting significantly modulate gene and protein expression involved in iron metabolism in leucocytes. In addition, the effects of exercise before and after starvation has been explored. The studied genes and proteins can be divided into those involved in import of iron into a cell and its storage (TFRC, FTL, FTH, PCBP1, PCBP2) and those responsible for its excretion like APP, FPN, PCBP3, PCBP4.

The data referring to changes in the expression of genes related to iron metabolism and intracellular proteins in leukocytes are limited [29]. To the best of our knowledge, our study is the first to evaluate levels of leukocytes’ genes and proteins related to iron metabolism in response to starvation in healthy individuals. Most of the studies associated with iron deficiency or iron metabolism were conducted in the context of anemia or immune system function and were based on serum markers [16,30]. Our study investigated the effect of starvation on iron homeostasis in leukocytes, and showed that *ferritins*, *APP*, *PCBP1* and *PCBP2* mRNAs rapidly decreased after fasting, while protein levels remained unchanged. Our results presenting *FTH*, *PCBP1* and *PCBP2* mRNA levels in response to starvation were similar to those described by Grzybkowska et al. (2019) where authors investigated changes in expression of the same genes in leukocytes of healthy individuals in response to a 100 km marathon [31]. The authors found no association between leukocytes gene expression, serum iron, and ferritins levels. Thus, energy deficiency caused by starvation and very intensive exercise showed similar changes in the mRNA levels of investigated genes in leukocytes [31]. On fission yeast it has been demonstrated that iron starvation may be associated with an decrease of stored iron and increased iron import into the cells [32]. In our study we observed a significant decrease in expression of *FTH* and *FTL* genes in leukocytes after 8 d of total starvation. One of the reasons of this changes could be related to reduction of labile iron pool (LIP) levels within cells [33] however the confirmation of this should be sought in direct determination of intracellular iron levels. In addition, IRE/IRP is the main system responsible for iron homeostasis in cells [34]. Under iron deficiency, IRP1 and IRP2 have a higher affinity to IRE regions, and their binding leads to the inhibition of *FTH* and *FTL* translation and protects TRFC against degradation Furthermore, observed increase in *TFRC* mRNA after starvation support this conclusion as it has been reported that activation of IRP leads to upregulation of the receptor as IRP protects *TFRC* mRNA from degradation [35]. Consistently APP which is involved in iron export from a cell was downregulated after starvation. IREs are the RNA stem loops that control ferritin translation and transferrin receptor mRNA stability. According to literature its presence has been also documented in APP transcript, and its IRP binding was reduced when LIP goes down what lead to reduction in *APP* translation [36]. Observed here significant decrease of *APP* mRNA after starvation indirectly points that it is possible that decrease LIP in this cells also decreased. Furthermore, IRE regions have been discovered in several transcripts of genes not associated with iron metabolism, such as: mitochondrial aconitase or hypoxia inducible factor 2 alpha (HIF2α) [24]. For example, during iron deficiency an inhibition of HIF2α and ferroportin mRNA translation was observed, with simultaneous stabilization of the proteins [37]. After 8 days of starvation we observed significant increase in *HIF-1 alpha* mRNA, which may indirectly indicate hypoxia conditions within cells. It has been shown that hypoxia significantly reduced transcription all ferritins in cultured lens epithelial cells, however this effect was stronger for *FTH* [38]. Our results indicated that *FTH* and *FTL* mRNA after starvation decreased comparably, and this difference could be associated with specificity of various types of cells. Moreover, similarly to our results, Goralska et al. (2014) observed the hypoxia-induced reduction in ferritin gene transcripts not corresponding to expression of ferritin proteins [38]. According to Huan et al. (2014), hypoxia had marginal influence on transcription *FTH*, but increased its translation through decreased IRP1-IRE interaction [39]. Furthemore, we observed significant increase in *FTRC* similar to Goralska et al. (2014), which also indicate that during fasting there was hypoxia conditions within WBC.

Because iron deficiency influences energy metabolism, mechanisms described above can lead to a significant inhibition of energy-consuming processes such as translation in cells and this can explain the discrepancy between transcript and protein levels. Significant increase in HIF-1a influence on energy production within cells. On a cellular level, reaction in which ATP is utilized are suppressed [40]. Literature data indicated that HIF-1a reduced lipolysis during fasting [41]. Moreover, experimental study conducted in vitro and in vivo (animal study) showed that HIF-1 alpha regulate both glucose uptake and glycolysis [42]. According to literature, not only HIF-1a but also or mainly HIF-2a is involved in iron homeostasis. *HIF-2a* mRNA contains IRE which indicates its dependence on iron level [24], however in the literature the data associated with HIF-2a are inconsistent. Fasting results in a hypoxic conditions and during hypoxia erythropoiesis, the major pathway of iron utilization, is stimulated [24]. Increased production of erythrocytes requires the delivery of iron to the bone marrow cells. Under normal conditions, iron absorption in enterocytes and iron recycling increased. It was well documented, that HIF-2a influences on iron absorption in enterocytes and thus plays a crucial role in maintaining iron balance in the organism. This transcriptional factor could directly regulating the transcription of the divalent metal transporter 1 (DMT1) and downregulated hepcidin expression, thereby increasing the absorption of iron in mice [43,44]. During prolonged fasting iron requirements must be met through recycling, mainly from tissue macrophages during erythrophagocytosis. However, the role of HIF in iron recycling was not fully confirmed. Mathieu et al. (2014) postulated that that neither HIF-1 nor HIF-2 are necessary to regulation the genes involved in iron recycling [45]. Moreover, in peripheral mononuclear blood cells (PMBC) Zhang et al. (2014) found enhancement of HIF-1α protein stability during iron deficiency with a simultaneous tendency to down-regulate HIF-2α translation [46]. It is possible that response to iron deficiency is cell-dependent and possible mechanisms require further research. It is possible that our findings showed the adaptive response of WBC to hypoxia conditions, oxidative stress and changes in intracellular iron level, which are closely related to each other. Moreover, here we observed that fasting induced significant rise in serum 8-isoprostane level which is a marker of lipid peroxidation. What indicates that oxidative stress plays a role in adaptation to fasting. Similarly increase in lipid peroxidation products has been observed after Ramadan fasting [47]. It is difficult to explain this phenomenon possibly lipid peroxidation products are released from adipose and other tissue during fasting.

Other genes of our interest were *PCBP1* and *PCBP2* which encode iron chaperone proteins but have different roles. PCBP1 is mainly involved in the transfer of iron to ferritin, while PCBP2 is involved in iron export from a cell [9]. Besides, PCBP1 and PCBP2 were demonstrated to form a complex for iron delivery to ferritin but also to some other proteins e.g., prolyl hydroxylase and asapragyl hydroxylase [48]. Interestingly, overexpression of PCBP1 enhanced and PCBP4 mitigate iron toxicity in yeast [9]. Here we observed decrease expression of *PCBP2* and *PCBP1,* and increase protein level of PCBP2 after fasting. PCBP2 binds to ferroportin and participates in the export of iron from the cell; it also binds to DTM1 and plays a role in the intracellular transport of iron or iron delivery to metalloenzymes [6,7]. However it is important to note that PCBP2 binds to ferroportin only when is loaded with iron [7] thus it can be expected that its function may differ dependent on the level of iron. Here we could not measure PCBP4 protein however our data shows that fasting leads to upregulation of its expression. The observations of the current study are difficult to interpret as we observed a decrease in *PCBP1* and *PCBP2* mRNA levels. This decrease could be associated with the use of transcripts for protein biosynthesis (a significant increase in PCBP2 levels was observed). Moreover, it is likely that gene transcription was inhibited during energy deficiency. Collectively, observations of the current study indicate that during fasting, mRNAs of genes associated with iron homeostasis are degraded in response to iron starvation. Physiological meaning of these changes remains to be studied. PCBP4 can protect from iron toxicity as mention above most of the data indicates that LIP decrease as a result of fasting. However, fasting can also induce autophagy of iron containing proteins thus iron trafficking what may enhance iron toxicity [49]. Conversely, iron deficiency has been shown to induce degradation of mRNA of mitochondrial Fe/S-cluster containing proteins what was crucial to protect against formation of non-functional mitochondria and oxidative stress. [50].

All of these data indicate that during starvation leucocytes adopt to scarcity of iron by lowering FTH, FTL and APP expression and increase expression of TFRC. The changes are probably trigger by decrease in LIP.

In the current study, fasting significantly reduced exercise performance in all study subjects. Increase in FTL levels 1 h after the exercise was the only detected difference in the levels of proteins involved in iron metabolism. FTL is mainly responsible for iron storage and its synthesis is controlled on both, transcriptional and translational levels. Previously, we have demonstrated that during stress, when stress activated protein kinases are functioning, ferritin undergoes partial degradation and releases iron [51]. Increase in iron levels affects the activity of iron-responsive proteins (IRP1 and IRP2) and leads to an increased translation of ferritin. It is not clear why only the FTL and not FTH levels increased after the exercise, and why the increase was transient. Conversely, exercise after 8 d of fasting did not affect the levels of any studied proteins. This difference could be associated with a reduced performed work after fasting or lower LIP level. Independently of the baseline levels, exercise elicited a decrease in FTH mRNA levels before and after fasting. Considering that FTL protein increase after the exercise it could indicates that after exercise stored iron increase at the expense of LIP. Exercise is associated with increase oxygen consumption and increase generation of reactive oxygen species (ROS) [52]. As LIP can potentiate ROS formation its reduction during exercise can limit oxidative stress.

## 4. Materials and Methods

### 4.1. Ethics

The study was approved by the Committee for Ethics in Scientific Research of Jan Dlugosz University in Czestochowa (Poland; KE-0/1/2019; 5 March 2019). The research project was performed respecting and applying the principles formulated in the World Medical Association Declaration of Helsinki—Ethical Principles for Medical Research Involving Human Subjects. All study participants have been informed about the purpose of the study and the associated risks of research methods used. Study participants had practiced fasting many times before. Their next voluntary fasting session was combined with the presented research. Qualified medical personnel informed the surveyed men about possible negative health effects of such practice. The subjects remained under medical care throughout the entire period of fasting. The participants gave their written, voluntary, and informed consent to participate in the study.

### 4.2. Study Group Characteristics

Thirteen volunteers, healthy men who had previously practiced starvation diet (for 3–42 d) participated in the study. The number of fasts previously held by the individual respondents ranged from 3 to 12. Each participant avoided starvation in the 6 months before the study. All participants underwent a medical examination and no contraindications to performing exhausting exercise were found. The subjects stated that they had practiced regular physical activity in the form of moderate-intensity yoga; their level of physical fitness was not controlled by the authors of the study. The inclusion criteria were: (1) previous experience in undertaking CWF of more than three days; (2) age range of 30–70; (3) body weight between 60–100 kg; (4) body mass index—BMI—20–29.9 kg/m^2^; (5) no chronic diseases; (6) systolic blood pressure in the range of 100–140 mmHg and diastolic blood pressure in the range of 60–90 mmHg. The Exclusion criteria were: (1) the presence of chronic diseases; (2) smoking cigarettes; (3) the use of medications, strong stimulants or psychoactive substances; (4) failure to complete the test procedure.

The basic somatic parameters of the participants are summarized in Table 1.

### 4.3. Anthropometrics

The participants, in good physical and mental health, arrived in the laboratory in the morning, after a 12-h fast and a night’s rest, having abstained from alcohol, medication, and exercise for the previous 2 d. At the beginning of the study, the subjects’ age and basic somatic data were recorded (body height, body weight, body fat content, FFM, TBW, and BMI), were determined via bioelectric impedance analysis using the Tanita TBF 300 A body composition analyzer (Tanita, Amsterdam, The Netherlands).

### 4.4. Exercise

The participants performed an exercise test of gradually increasing intensity until the individual maximum exercise capacity was reached (until exhaustion) using Excalibur Sport cyclo ergometer (Lode B.V. Groningen, The Netherlands). The initial load was 60 W, gradually increasing by 30 W every 3 min. The exercise was stopped when the subject was unable to maintain the set pedaling rhythm; when the oxygen uptake began to stabilize at its maximum (or decreased); or when the heart rate did not increase or stabilized at the maximum level (or began to decrease). After 8 d of complete fasting, during which the subjects only consumed ad libitum mineral water with an average ionic content, the procedures described above were repeated.

### 4.5. Blood Sampling

For biochemical and gene expression analyses, 10 mL of blood was collected from the venous vein. For Hb, Hct, β-hydroxybutyrate (enzymatically using the RANDOX RANBUT diagnostic kit (Randox Laboratories Ltd., Crumlin, UK)), and uric acid level (enzymatic methods using ABBOTT’s ARCHITECT c SYSTEM 4000 analyzer) determinations, the blood was collected twice: at baseline (before the first exercise) and after 8 d of starvation (before the second exercise).

To assess leukocyte gene expression and intracellular protein levels, the blood was collected at three time points: before each exercise (before and after starvation), and 3 min and 3 h after the test to refuse of work. Immediately after collection, 4 mL of the whole blood were mixed with 20 mL of the red blood cell lysis buffer (RBCL, A&A Biotechnology, Gdynia, Poland), and incubated on ice for up to 20 min. The samples were then centrifuged (4 °C, 3000× *g*, 10 min), and the obtained leukocyte pellet was divided in two (for mRNA and proteins analysis), and processed as described in Section 4.6 and Section 4.8 (accordingly).

### 4.6. Determination of Serum 8-Isoprostane

Serum 8-Isoprostane concentration were determined using an ELISA kit (Cayman Chemicals, Ann Arbor, MI, USA) as instructed by the manufacturer.

### 4.7. RNA Analysis

RNA was isolated as described by Chomczyński and Sacchi (1987). Briefly, the leukocytes were lysed in 800 µL of Fenozol (A&A Biotechnology, Gdynia, Poland). After 10-min incubation at 50 °C to separate the aqueous phase containing RNA, 200 µL of chloroform were added. Next, the samples were centrifuged (4 °C, 12,000× *g*, 30 min) and the aqueous phase was collected. RNA was precipitated by the addition of 500 µL of isopropanol. After 15-min incubation at room temperature (22 °C), the samples were centrifuged again (4 °C, 12,000× *g*, 15 min). The obtained pellet was rinsed in 1 mL of 75% ethanol and centrifuged (4 °C, 7500× *g*, 5 min). After pouring out the ethanol and drying, the pellet was suspended in 25 µL of water. The RNA quantity and quality were checked using a photometer (EppendorfBioPhotometer Plus, Eppendorf AG, Hamburg, Germany). For all samples, the A230/A260 and A230/A320 read ratios were over 1.7. Applied procedure was previously described [31].

### 4.8. Reverse-Transcription and Quantitative Real-Time Polymerase Chain Reaction (RT-qPCR)

For the analysis, 1000 ng RNA was reverse-transcribed using the Transcriptor Kit (Roche, Warsaw, Poland) with oligo-dT primers. Gene expression was analyzed by RT-qPCR (Agilent, Aria, Poland). Each sample was analyzed in triplicate. Polymerase (Agilent, Warsaw, Poland) was used on Eppendorf Mastercycler Gradient 5331.

The time-temperature profile of the reaction was consistent with the manufacturer’s instructions. A melt curve analysis was performer after each reaction. Tubulin beta class I, NM_001293213 (*TUBB*) was used as a housekeeping gene. The following specific primers were used (Primer 3 Web tool) (Table 2):

### 4.9. Immunoblotting

The leukocytes were prepared as described in Section 4.5, and resuspended and homogenized on ice in RIPA Lysis Buffer (VWR Live Science, N653, Randor, PA, USA). The samples were then incubated for 40 min on ice with gentle shaking. The cell lysates were cleared by centrifugation at 17,000× *g* for 20 min. Protein concentration in the lysates was determined using a Bradford method [53] and samples containing equal amounts of protein were heated to 95 °C for 10 min in Laemmli buffer (Bio-Rad, 161-0747, Hercules, CA, USA). Protein lysates were resolved by electrophoresis on 4–20% Mini-PROTEAN TGX stain- free gels (Bio-Rad, 4568093). Protein lysates were resolved by electrophoresis on 4–20% Mini-PROTEAN TGX stain- free gels (Bio-Rad, 4568093). Stain-free gels contains trihalo compound, which is covalently bound to tryptophan residues and make proteins fluorescent directly in the gel after a short photoactivation in UV light, allowing the immediate visualization of proteins. For these reason, immediately after separation, the gels were activated under Bio-Rad UV transilluminator (Chemi Doc MP). The proteins were then transferred onto a membrane by using a semi-dry technique, using Bio-Rad Trans-Blot Turbo System. Once the blotting step was complete, all separated proteins were visualized using the Chemi Doc MP system. To verify an equal protein loading, all bands in each lane were taken for normalization. Due to the large size of the entire membrane, the figure shows a section of the membrane within the range of the tested protein. The membrane segment reflects the protein loading from the whole membrane. After blocking in 5% (*w*/*v*) solution of dried skim milk in TBS buffer containing 0.1% (*v*/*v*) Tween 20, the membrane was incubated with specific primary antibodies, overnight at 4 °C. The following primary, anti-rabbit antibodies (1:1000 dilution) were used: anti-SLCA40A1 (abcam, ab78066, Cambridge, UK); anti-hnRNP E1 (Cell Signaling Technology, 8534, Denver, CO, USA); anti-hnRNP E2 (Cell Signaling Technology, 83017, Denver, CO, USA); anti-FTH1 (Cell Signaling Technology, 3998, Denver, CO, USA); and anti-Ferritin Light Chain (abcam, ab69090, Cambridge, UK). All antibodies have been validated in previous cell line studies. The membrane was then incubated with anti-rabbit secondary antibody (1:20 000 solution) (Sigma Aldrich, A 9169, Saint Louis, MO, USA). The immunoreactive bands were visualized using enhanced chemiluminescence and Chemi Doc MP imaging system. Changes in protein levels were normalized to total protein load in each lane using Image Lab Software (Bio-Rad).

### 4.10. Statistical Analysis

Basic statistical analyses of all parameters were performed, and the mean values and SEM were calculated. To check the normality of the distribution, Shapiro–Wilk test was used. To compare the values at the same time points before and after starvation or before and after exercise, paired *t*-test was used. Additionally, two-way ANOVA was performed to determine the effects of fasting and exercise. To calculate the effect size, Cohen’s d was used. Standardized effects were classified as small (>0.2), moderate (>0.5), and large (>0.8). All calculations were performed in GraphPad Prism 6.0 (www.graphpad.com, accessed date 19 March 2021).

Gene expression was calculated as a comparative Ct method described by Schmitten and Livak (2008). Housekeeping gene TUBB was selected experimentally. In order to calculate relative expression, we measured Ct values of target and housekeeping genes. The difference between the threshold cycles was displayed as binary logarithm (log2). The relative expression was calculated in Microsoft Excel 2010. After data transformation to linear values, and statistical significance was evaluated using the Shapiro–Wilk test to assess for normal distribution and the Wilcoxon test for comparison of results before and after fasting. All calculations and graphics were performed using GraphPad Prism 6.0. Differences were considered statistically significant at a level of *p* < 0.05.

To determine changes in blood and plasma volume, Dill and Costill [54] formula was used:

BV_A_ = BV_B_ (Hb_B_/Hb_A_)

CV_A_ = BV_A_ (HCT_A_)

PV_A_ = BV_A_ ─ CV_A_

Δ BV, % = 100 (BV_A_ ─ BV_B_)/BV_B_

Δ PV, % = 100 (PV_A_ ─ PV_B_)/PV_B_

## 5. Conclusions

In conclusion, our data revel that fasting induce significant changes in genes expression and only small changes in proteins involved in iron metabolism in human leukocytes. Observed decrease in genes involved in iron storage and export and increase in those responsible for iron import into a cell resemble those data when only iron deficiency was applied. Conversely, we did not observe significant changes in proteins like ferritin H, L and ferroportin after 8 d of fasting what has been shown in iron deficiency state induced e.g., by iron chelator [51]. Certainly, more study is needed to fully understand the effects of fasting on iron metabolism. As mention above iron is very important for the most of physiological function of any cell and understanding how its metabolism reacts to fasting in crucial. Based on the above observations, it can be assumed that the human body, despite the lack of exogenous iron, is able to activate compensatory mechanisms on the cellular level to enable iron acquisition from the internal stores.

### Study Limitation

Our study has some limitations: We investigated changes in mRNA and proteins only in WBC. Our results concern only healthy men adapted to starvation.

## Figures and Tables

**Table 1:** Basic somatic and biochemical statistics of the study group before and after 8 d of fasting.

*n* = 13	Baseline Values before Fasting		Baseline Values after Fasting		Δ(Before−after)		Cohen’s d (Effect Size)
Parameter	Mean	SD	Mean	SD	Mean	SD	
Age	50.3 ± 14.2						
Body mass (kg)	79.38	10.79	73.43 *	10.26	–5.96	0.79	0.57
Fat mass (kg)	15.28	5.42	13.21 *	5.33	–2.07	0.56	0.39
FFM (kg)	64.11	5.87	60.07 *	5.88	–4.04	0.89	0.69
TBW (kg)	46.94	4.29	44.08 *	4.11	–2.86	0.62	0.68
BMI (kg/m^2^)	24.89	3.52	23.02 *	3.35	–1.87	0.25	0.54
β-hydroxybutyrate (mmol/L)	0.32	0.22	4.93 *	0.65	3.74	1.94	9.5
Hb (g%)	15.43	0.91	14.47	1.24	–0.39	1.83	0.88
HCT (%)	45.4	2.80	43.5	3.66	–1.09	5.56	0.58
PV (%)	100	-	98.54	7.06	−1.46	7.06	0.28
BV (%)	100	-	104.00	12.99	0.84	7.37	−0.42

Data are mean ± SE (n = 13) * *p* = 0.0001, significantly different as compared to before fasting by *t*-test; FFM, fat-free mass; TBW, total body water; BMI, body mass index; Hb, hemoglobin; Hct, hematocrit, PV, plasma volume, BV, blood volume.

**Table 2:** Listing of primer sequences used for qRT-PCR.

Gene	Forward Primer (5′–3′)	Reverse Primer (5′–3′)
*TUBB* NM_001293213	TCCACGGCCTTGCTCTTGTTT	GACATCAAGGCGCATGTGAAC
*PCBP1* NM_006196	AGAGTCATGACCATTCCGTAC	TCCTTGAATCGAGTAGGCATC
*PCBP2* NM_001128913	TCCAGCTCTCCGGTCATCTTT	ACTGAATCCGGTGTTGCCATG
*PCBP3* NM_020528.3	TGTGAAGAAGATGCGTGAGG	RGCTGTTGCTATGGAGTTGA
*PCBP4* NM_033010.2	CCTGTCTAGAGACGGCCAAG	AGCAGGTGGTAAAGCCAAGA
*FTH1* NM_002032	TCCTACGTTTACCTGTCCATG	CTGCAGCTTCATCAGTTTCTC
*FTL* NM_000146	GTCAATTTGTACCTGCAGGCC	CTCGGCCAATTCGCGGAA
*TFRC* NM_001128148	TGCAGCAGTGAGTCTCTTCA	AGGCCCATCTCCTTAACGAG
*APP* NM_000484.4	TCAGTCTCTCTCCCTGCTCT	GGTGGTTTTCGTTTCGTTTCGGTCA

## Data Availability

The data that support the findings of this study are available from the corresponding author upon reasonable request.

## Abbreviations

APP	amyloid precursor protein
BMI	body mass index
BV	blood volume
DMT1	divalent metal transporter 1
FFM	fat-free mass
FTH	ferritin heavy chain
FTL	ferritin light chain
FPN	ferroportin
Hct	hematocrit
Hb	hemoglobin
IRP 1-2	iron regulatory proteins 1-2
LIP	labile iron pool
PCBP 1-4	poly(rC)-binding protein 1-4
PV	plasma volume
TBW	total body water;
TFRC	transferrin receptor
WBC	white blood cells
